# Consequences of COVID19-pandemic lockdown on Italian occupational physicians’ psychosocial health

**DOI:** 10.1371/journal.pone.0243194

**Published:** 2021-02-03

**Authors:** Simone De Sio, Giuseppe La Torre, Giuseppe Buomprisco, Ekaterina Lapteva, Roberto Perri, Paola Corbosiero, Pietro Ferraro, Arianna Giovannetti, Emilio Greco, Fabrizio Cedrone

**Affiliations:** 1 R.U. of Occupational Medicine, “Sapienza” University of Rome, Rome, Italy; 2 Department of Public Health and Infectious Diseases, “Sapienza” University of Rome, Rome, Italy; 3 Department of Prevention, ASL Foggia, Foggia, Italy; 4 INPS, Italian Institute of Social Welfare, Rome, Italy; 5 Link Campus University, Rome, Italy; 6 Postgraduate School of Hygiene and Preventive Medicine, University “G. d’Annunzio” of Chieti-Pescara, Chieti, Italy; Universita Cattolica del Sacro Cuore Sede di Roma, ITALY

## Abstract

COVID-19 was declared a pandemic on March 12, 2020. Italy has been the most affected country in the world, right after China. Healthcare workers (HCWs) were among the hardest hit by this event from both a working and psychological point of view. The aim of this web-based cross-sectional study is to assess the consequences of the COVID-19 pandemic on Italian Occupational Physicians’ well-being and psychological distress, in relation to demographic and occupational characteristic, lifestyle and habits during the lockdown period. We conducted a web-based cross-sectional survey questionnaire from April 1 to April 21^st^, 2020. To evaluate the level of psychological distress and the level of well-being, the general Health Questionnaire-12 (GHQ-12) and the WHO-5 Wellbeing Index were utilized. Since the statistical assumptions were respected, we proceeded with an analysis of variance (ANOVA) to ascertain the differences between the averages of the scores of the GHQ-12. Doctors who live in the most affected regions have a prevalence of psychological distress higher than their colleagues from the rest of Italy. ANOVA shows significant differences relating to the female gender, and to the life changes provoked by the lockdown for example not feeling sheltered at home or suffering from loneliness. This study showed a high prevalence of psychological distress in occupational physicians. To prevent the occurrence of mental disorders among Occupational Physicians, it is urgent to put in place policies of psychological support and well-being preservation.

## Introduction

In January 2020, the World Health Organization (WHO) declared the outbreak of the new coronavirus epidemic (COVID-19) to be a Public Health Emergency of International Concern. COVID-19 was declared pandemic on March 12, 2020, and on the same day there were 15,113 people infected and 1,016 deaths in Italy. Subsequently, on March 27, 2020, Italy the most affected became the country in the world by the spread of coronavirus, counting over 86,000 confirmed cases. On April 1^st^, Italy counted 28,403 hospitalizations of which 4,035 in intensive care [1].

Healthcare workers (HCWs) were among the hardest hit by this event from both a working and psychological point of view.

The contagion and the burden on health services was not distributed evenly across the national territory but affected mostly the regions of northern Italy, with areas such as Lombardia, Piemonte, Veneto and Emilia-Romagna, for a total of 72.110 cases; in the remaining 16 Italian Regions were registered 35.559 cases (April 21^st^, 2020) [2].

On April 29^th^, 2020, the Italian Ministry of Health published a paper dealing with the “Operational indications relating to the activities of Occupational Physicians (OPs) in the context of measures to contend and contain the spread of the SARS-CoV-2 virus in the workplace and in the community”. In this document, the role of the occupational physician had a central position in the anti-contagion measures, in the active surveillance of workers and in all the preventive activities aimed at guaranteeing the protection of worker’s health upon the resumption of working activities. OPs have been invited to identify and report to employers, any situation of weakness among company personnel and to manage the reintegration of subjects with previous COVID-19 infection.

Several studies have shown how severely the well-being of Physicians can be affected in pandemic periods. In fact, during the previous SARS epidemic of 2003 significant psychosocial effects were reported on HCWs and experiences with very stressful challenges that can trigger common mental disorders, including anxiety and depression, that can, ultimately, result in hazards that exceed the consequences of the COVID-19 pandemic itself [3–7].

Recently, a meta-analysis has studied a population of physicians engaged in the management of new viral outbreaks, investigating its psychological aspects. It has shown how young Physicians with little working seniority, being parents, and experiencing a long quarantine suffered an important psychological distress [8].

In addition, lockdown measures (closing of schools, universities, all non-essential businesses and parks, social distancing, limitation of movements and transport, etc.), imposed by many governments, including the Italian one (the “lockdown” was introduced in Italy on March 9^th^, 2020) in order to slow down the spread of the virus, resulted in a reduced perception of health and an increased feeling of distress in the general population, even if there is a lack of studies that explore this relationship [9].

In this dramatic national scenario, the figure of the Occupational Physician was important in all the essential businesses (Hospitals at first) to implement anti COVID-19 prevention measures in the workplaces and in the active health surveillance of workers, during the lockdown.

The aim of this web-based cross-sectional study is to assess well-being and psychological distress on Italian occupational physicians’, in relation to demographic and occupational characteristics, lifestyle and habits during the lockdown period.

## Materials and methods

### Study design and participants

We conducted a web-based cross-sectional survey based on Google^®^ Forms to collect data. The participation was available across the lockdown period that started in Italy on March 9, 2020 and it was voluntary and anonymous. The link of the survey was published on the first author’s personal website (https://sites.google.com/a/uniroma1.it/simonedesio) and was sent to 283 Italian OPs subscribed to the mailing list of the Research Unit of Occupational Medicine of “Sapienza” University of Rome.

### Data collection

The participants answered the questionnaire from April 1 to April 21, 2020. The questionnaire consisted of three sections which investigated: 1) demographic and occupational variables, 2) lifestyle and habits variables, 3) psychological distress and perceived well-being.

### Ethical statement

This study was conducted in conformity with the Declaration of Helsinki. An electronic informed consent was obtained from each participant before the start of the investigation.

The research involved the use of completely anonymous surveys and the participants were not considered as "vulnerable". The participation was voluntary, and it didn’t induce undue psychological stress or anxiety. For these reasons, no ethical approvement has been requested, as required by the institutional review board (IRB) of Sapienza University; a self-certification was provided about the respect of ethical principles.

### Questionnaire sections

#### Demographic and occupational variables

The first section of the survey explored demographic and occupational characteristics. The demographic variables included gender (male or female), age and marital status (single or cohabiting). Occupational variables included: 1) working area, in relation to the Italian regions most affected by COVID-19 (Veneto, Lombardia, Piemonte and Emilia-Romagna) and those which were less affected; 2) job seniority; 3) night shift work; 4) use of “smart-working” (also intended as “telework” or “remote working”); 5) availability and use of personal protective equipment; 6) changes in job demand.

#### Lifestyle and habits changes

The second section of the survey explored lifestyle and habits variables. Lifestyle variables included living alone or cohabiting (with partner, family or friend/roommates), feeling sheltered at home, suffering loneliness, feeling comfortable at home. Habits variables included smoking, eating habits, and alcohol consumption.

#### Psychological distress and perceived well-being

The third section of the survey consisted of two questionnaires: the 12-item version of the General Health Questionnaire (GHQ-12) to evaluate psychological distress and the 5-item World Health Organization Well-Being Index (WHO-5) to explore subjective well-being.

The 12-item General Health Questionnaire (GHQ-12) is a self-report indicator of psychiatric disorders currently experienced by the responder with respect of the last two weeks [10]. It consisted of 12 questions with 4 possible answers: 1) less than usual, 2) no more than usual, 3) rather more than usual or 4) much more than usual, according to how much the symptoms indicated were experienced. Two scoring methods can be applied: a dichotomous one (0-0-1-1), suggested by the original authors, or a Likert- type (0-1-2-3) [11]. We have opted for the first approach, to ensure less dispersion in the results, considering a score ≥4 as an indicator of psychological distress.

The WHO-5 items questionnaire is a short and generic rating scale checking subjective well-being [12], chosen because it is short, simple and has been used to study the well-being of workers both in Italy and worldwide [13–15]. It consists of 5 positively worded questions, rated by the respondent from 0 to 5, with higher scores indicating better conditions; a score below 13 indicates poor well-being.

### Statistical analysis

Quantitative variables were expressed as median and interquartile range (IQR) or as mean and standard deviation in relation to their distribution, qualitative variables were indicated as frequency and percentage. Univariate analysis, including chi-square for categorical variables was conducted to assess differences between groups of descriptive variables and the outcome of the questionnaires (dichotomous). The analysis of kurtosis showed that the GHQ-12 questionnaire had a normal distribution in each of the groups formed, according to the answers given. Furthermore, using the *Levene's Test*, centred using the 10% trimmed mean, the homogeneity of variances has been demonstrated. Since the statistical assumptions were respected, we proceeded with an analysis of variance (one-way ANOVA) to analyse the differences between the averages of the scores of the GHQ-12 questionnaire between groups identified by the answers given. A post-hoc *Tukey test* was subsequently performed to demonstrate which specific groups were significantly different from each other. As the score of the WHO-5 questionnaire did not satisfy the assumptions for the analysis of variance (one-way ANOVA), it was not carried out and was used to distinguish one group with poor well-being and another with a good level of well-being, useful both for the descriptive univariate and as an independent variable of the one-way ANOVA. Statistical significance was set at *P*<0.05. The data was analysed using the statistical software Stata® version 15.

## Results

A total of 202 participants completed the questionnaires (Response rate of 71,4%). Whereas the population of OPs in Italy is approximately 7400 units, the sample size achieved is consistent with a margin error of 7% and a confidence level of 95%. These results demonstrate adequate sample size of the study. All the results about prevalence and univariate analysis are shown in Table 1.

**Table 1:** Sample characteristics.

	*n* (%)	Psychosocial Distress	*P-*value	Poor well-being	*P*-value
***I*. *Demographic variables***					
**Total**	202 (100.00)	180 (89.11)		93 (46.04)	
**Gender**					
Female	79 (39.11)	72 (91.14)	0.458	44 (55.70)	0.027
Male	123 (60.89)	108(87.80)		49 (39.84)	
**Age median (IQR)**	51 (44–62)	50.5 (44.62)	0.709	47 (42–60)	0.062
**Age cat**					
≤50	100 (49.50)	90 (90.00)	0687	54 (54.00)	0.025
>50	102 (50.50)	90 (88.24)		39 (38.24)	
**Marital Status**					
Single	26 (12.87)	25 (96.15)	0.217	14 (53.85)	0.392
Cohabiting	176 (87.13)	155 (88.07)		79 (44.89)	
**Italian working Region**					
Most affected	59 (29.21)	57 (96.61)	**0.028**	36 (61.02)	0.006
Less affected	143 (70.79)	123 (86.01)		57 (39.86)	
***II*. *Job characteristics***					
**National Health System Staff**	49 (24.26)	46 (93.88)	0.218	25 (51.02)	0.422
**Freelancers**	153 (75.74)	134 (87.58)		68 (44.44)	
**Job Seniority, median (IQR)**	18 (10–30)				
≤18	108	98 (90.74)	0.425	58 (53.70)	0.019
>18	94	82 (87.23)		35 (37.23)	
***III*. *Lockdown/pandemic variables***					
**Smart working**					
Yes	86(42.57)	76 (88.37)	0.778	34 (39.53)	0.110
No	116 (57.43)	104 (89.66)		59 (50.86)	
**Night shifts**					
Yes	2(0.99)	2 (100)	0.619	0 (0.00)	0.501*
No	200 (99.01)	178 (89.00)		93 (46.50)	
**Job demand during pandemic**					
Higher than before	74(36.63)	63 (85.14)	0.346	27 (36.49)	0.052
Lower than before	101(50.00)	93 (92.08)		55 (54.46)	
Unchanged	27(13.37)	24 (88.89)		11 (40.74)	
**Use of Personal protective equipment (PPE)**					
Yes	180 (89.11)	161 (89.44)	0.661	82 (45.56)	0.693
No	22 (10.89)	19 (86.36)		11 (50.00)	
**Cohabitants during the lockdown**					
Alone	25 (12.38)	24 (96.00)	0.566*	13 (52.00)	0.307
Partner	49 (24.26)	43 (87.76)		18 (36.73)	
Family	128 (63.37)	114 (88.28)		62 (48.44)	
**Feeling sheltered at home**					
Yes	48 (23.76)	36 (75.00)	0.001*	4 (8.33)	<0.001*
No	58 (28.71)	57 (98.28)		44 (75.86)	
Sometimes	96 (47.52)	87 (90.63)		45 (46.88)	
**Suffering loneliness**					
Yes	21 (10.40)	21 (100)	0.247*	20 (95.24)	<0.001*
No	156 (77.23)	137 (87.82)		63 (40.38)	
Sometimes	25 (12.38)	22 (88.00)		10 (40.00)	
**Feeling comfortable at home**					
Yes	129 (63.86)	109 (84.50)	0.020*	49 (37.98)	0.007
No	13 (6.44)	13 (100)		9 (69.23)	
Sometimes	60 (29.70)	58 (96.67)		35 (58.33)	
***IV*. *Lifestyles habits and changing***					
**Smoke habits**					
Smokers	35 (17.33)	30 (85.71)	0.549	16 (45.71)	0.966
Not smokers	167 (82.67)	150 (89.82)		77 (46.11)	
**Number of cigarettes, median (IQR)**	8 (5–15)				
≤8	23 (11.38)	20 (86.96)	1.000	10 (43.48)	0.713
>8	12 (5.94)	10 (83.33)		6 (50.00)	
Smoking more during lockdown	12 (5.94)	11 (91.67)	1.000	4 (33.33)	0.476
Smoking less during lockdown	23 (11.38)	20 (86.96)		12 (52.17)	
**Changed eating habits**					
Yes	88 (43.56)	78 (88.64)	0.850	52 (59.09)	0.001
No	114 (56.44)	102 (89.47)		41 (35.96)	
Increasing in food intake	61 (69.32)	53 (86.89)	0.437	37 (60.66)	0.654
Decreasing in food intake	27 (30.68)	25 (92.59)		15 (55.56)	
**Increased alcohol consumption**					
Yes	24 (11.88)	23 (95.83)	0.260*	13 (54.17)	0.395
No	178 (88.12**)**	157 (88.20)		80 (44.94)	
**Poor well-being (WHO-5)**	93 (46.04)	90 (96.77)	0.001*	-	-
**High wellbeing (WHO-5)**	109 (53.96)	90 (82.57)		-	

### Demographic and occupational variables

Regarding demographic and occupational variables, the total sample was made of:

1. 79 (39.11%) females and 123 (60.89%) were males, the median age of the specimen was 51 years (IQR: 44–62);
2. 59 (29.21%) worked in the Italian Regions with the higher number of COVID-19 cases (Veneto, Lombardia, Piemonte and Emilia-Romagna), and 143 (70.79%) worked in the other Italian Regions;
3. Median Job Seniority was 18 years (IQR: 10–30);
4. 86 (42.57%) OPs who were experiencing “smart-working”;
5. 180 (89.11%) declared use of personal protective equipment;
6. 74 (36.63%) working more than before the pandemic, 101 (50.00%) working less and 27 (13.37%) unchanged;
7. 49 (24.26%) declared to be National Health Service employees and 153 (75.74%) declared to be freelancers.

### Lifestyle habits changes

Regarding lifestyle, the total sample consisted of:

1. 48 (23.76%) “feeling sheltered at home”, 21 (10.40%) “suffering from loneliness” and 129 (63.86%) “feeling comfortable at home”;
2. 35 (17.33%) smokers with a median of 8 (IQR 5–15) cigarettes smoked per day, of them 12 (5.94%) claimed to have increased cigarette consumption during lockdown;
3. 88 (43.56%) changed eating habits and 61 (69.32%) increased food intake;
4. 24 (11.88%) reported having increased alcohol consumption.

### Psychological distress and perceived well-being

The evaluation by the GHQ-12 demonstrated the prevalence of psychological distress:

1. higher in females than males (91.14% vs 87.80%, *P* = 0.458);
2. higher in participants working in the most affected Regions than those from the least affected ones (96.61% vs 86.01%, *P* = 0.028);
3. higher in Ops who “don’t feel sheltered in their home” than those who “feel sheltered in their home” (98.28% vs 75.00%, *P* = 0.001);
4. higher in OPs who “suffer loneliness” than those who “don’t suffer loneliness” (100.00% vs 87.82%, *P* = 0.001);
5. higher in OPs who “don’t feel comfortable at the home” than those who “feel comfortable at home (100.00% vs 84.50%, *P* = 0.020);
6. higher in OPs who reported poor well-being at WHO-5 than others (96.77% vs 82.57%, *P* = 0.001).

The evaluation by the WHO-5 demonstrated the prevalence of poor well-being:

1. in 46.04% of total specimens, higher in females than in males (55.70% vs 39.84%, *P* = 0.027);
2. higher in those aged ≤50 than in those aged >50 (54.00% vs 38.24%, *P* = 0.025);
3. higher in participants working in the most affected Regions than those from the least affected ones (61.02% vs 39.86%, *P* = 0.006);
4. higher in those with a lower job seniority than others (53.70% vs 37.23%, *P* = 0.019);
5. higher in OPs who “don’t feel sheltered in their home” than those who “feel sheltered in their home” (75.86% vs 8.33%, *P*<0.001);
6. higher in OPs who “suffer loneliness” than those who “don’t suffer loneliness” (95.24% vs 40.38%, *P*<0.001);
7. higher in OPs who don’t feel comfortable at the home” than those who “feel comfortable at home (69.23% vs 37.98%, *P* = 0.007);
8. higher in OPs who changed their eating habits than those who did not (59.09% vs 35.96, *P* = 0.001).

The analysis of variance (one-way ANOVA) demonstrated:

1. differences between males and females (F *=* 4.82, *P* = 0.029) with a contrast of -0.75 in GHQ-12 score (standard error = 0.34);
2. differences in respect of the statement "*feel sheltered at home*" (F = 22.52, *P*<0.001), in this case, a *Tukey post-hoc test* revealed that mean GHQ-12 score differ between “no” vs “yes” (2.82 ± 0.42 GHQ-12 score, *P*<0.001), between who answered “*sometimes*” vs “*yes*” (1.15 ± 0.38 GHQ-12 score, *P* = 0.009) and “*sometimes*” vs “*no*” (-1.66 ± 0.36 GHQ-12 score, *P*<0.001);
3. differences in respect of the statement “*suffer from loneliness*" (F = 7.93, *P*<0.001), in this case, a *Tukey post-hoc test* revealed that mean GHQ-12 score differ between “no” vs “yes” (-2.13 ± 0.54 GHQ-12 score, p<0.001), between who answered “*sometimes*” vs “*yes*” (-1.54 ± 0.69 GHQ-12 score, *P* = 0.067) and “*sometimes*” vs “*no*” (0.58 ± 0.50 GHQ-12 score, *P* = 0.478);
4. differences in respect of the statement "*feel comfortable at home*” (F = 11.16, *P*<0.001), in this case a *Tukey post-hoc test* revealed that mean GHQ-12 score differed between “*sometimes*” vs “*yes*” (1.67 ± 0.36 GHQ-12 score, *P*<0.001), between who answered “*no*” vs “*yes*” (1.05 ± 0.67 GHQ-12 score, p = 0.260) and “*sometimes*” vs “*no*” (0.62 ± 0.70 GHQ-12 score, *P* = 0.651).

Results of ANOVA analysis of the mean GHQ-12 scores and *Tukey post-hoc test* are shown, respectively, in Tables 2 and 3.

**Table 2:** One-way Anova of the General Health Questionnaire-12 score.

	General Health Questionnaire-12			
	Means (SD)	F	df	*P*-value
***I*. *Demographic variables***				
**Gender**				
Female	7.05 (2.53)	4.82	1	**0.029**
Male	6.29 (2.30)			
**Age**				
≤50	6.81 (2.55)	1.66	1	0.199
>50	6.37 (2.26)			
**Marital Status**				
Single	7.38 (2.22)	3.27	1	0.072
Married	6.47 (2.42)			
**Italian working Region**				
Most affected	6.52 (2.63)	0.35	1	0.555
Less affected	6.74 (1.77)			
***II*. *Job characteristics***				
**National Health System Staff**	7.02 (2.13)	2.07	1	0.152
**Freelancers**	6.45 (2.49)			
**Job Seniority, median (IQR)**				
≤18	6.87 (2.48)	3.39	1	0.067
>18	6.25 (2.30)			
***III*. *Lockdown/pandemic variables***				
Smart working	6.65 (2.39)	0.10	1	0.754
Not in Smart working	6.54 (2.44)			
**Job demand during pandemic**				
Higher than before	5.88 (2.39)	1.59	2	0.207
Minor than before	6.81 (2.38)			
Unchanged	6.54 (2.45)			
**Attending videoconferences more often than before lockdown**				
Yes	6.55 (2.28)	0.10	1	0.751
No	6.66 (2.67)			
**Use of Personal protective equipment (PPE)**				
Yes	6.56 (3.37)			
No	6.77 (2.82)	0.14	1	0.707
**Cohabitants during the lockdown**				
Alone	7.2 (2.06)	0.94	2	0.393
Partner	6.57 (2.50)			
Family	6.47 (2.44)			
**Feeling sheltered at home**				
Yes	5.22 (2.06)	22.52	2	**<0.001**
No	8.05 (2.53)			
Sometimes	6.38 (3.02)			
**Suffering loneliness**				
Yes	8.42 (1.91)	7.93	2	**<0.001**
No	6.29 (2.32)			
Sometimes	6.88 (2.69)			
**Feeling comfortable at home**				
Yes	6.02 (2.31)	11.16	2	**<0.001**
No	7.07 (2.39)			
Sometimes	7.7 (2.24)			
***IV*. *Lifestyles habits and changing***				
**Smoke habits**				
Smokers	6.8 (2.79)	0.32	1	0.572
Not smokers	6.54 (2.33)			
**Number of cigarettes**				
≤8	6.43 (2.62)	1.15	1	0.291
>8	7.5 (3.08)			
Smoking more during lockdown	7.25 (2.49)	0.42	1	0.522
Smoking less during lockdown	6.60 (2.91)			
**Changing in eating habits**				
Yes	6.76 (2.73)	0.79	1	0.375
No	6.45 (2.14)			
Increasing in food intake	6.57 (2.78)	0.94	1	0.336
Decreasing in food intake	7.18 (2.61)			
**Increasing in alcohol consumption**				
Yes	7.04 (2.72)	0.95	1	0.330
No	6.52 (2.37)			
**Poor well-being (WHO-5)**	7.73 (2.27)	47.32	1	**<0.001**
**High wellbeing (WHO-5)**	5.61 (2.09)			

**Table 3:** Tukey post-hoc test of the General Health Questionnaire-12 score.

General Health Questionnaire-12			
	Contrast	Standard Error	*P*-value
***I*. *Demographic variables***			
**Gender**			
Male vs Female	- 0.75	0.34	0.029
**Feeling sheltered at home**			
No vs yes	2.82	0.42	<0.001
Sometimes vs yes	1.15	0.38	0.009
Sometimes vs no	-1.66	0.36	<0.001
**Suffering loneliness**			
No vs yes	-2.13	0.54	<0.001
Sometimes vs yes	-1.54	0.69	0.067
Sometimes vs no	0.58	0.50	0.478
**Feeling comfortable at home**			
No vs yes	1.05	0.67	0.260
Sometimes vs yes	1.67	0.36	<0.001
Sometimes vs no	0.62	0.70	0.651
**Well-being vs Poor well-being**	-2.11	0.30	<0.001

## Discussion

This study focused on occupational physicians, assessing their psychological distress, perceived well-being and lifestyle changes during the lockdown caused by the COVID-19 pandemic.

As previously demonstrated, COVID-19 pandemic and subsequent lockdown measures had a strong impact on doctors’ wellbeing and mental health [16]. Many studied carried out in Italy during this period, also demonstrated the negative effects of the quarantine on people’s mental and physical health. Increasing violence, worsening of pre-existing psychiatric illness and of physical health in patients with comorbidities, represented an additional source of stress for the medical category [17–20].

The General Health Questionnaire (12 items version) derives from an original version consisting of 60 items developed by Goldberg and colleagues in 1978. Many different shortened versions are available and all of them have been validated [21]. The 28-item version has been successfully used in the past to assess HCWs psychological distress during the H1N1 influenza pandemic [22].

It cannot indicate a specific diagnosis (depression, anxiety, etc.), but it represents a general indicator of distress and/or potential problems. This tool has previously demonstrated to have a good psychometric value and to be able to assess work-related stress amongst HCWs [23, 24].

What clearly emerged from this web-survey, is the high prevalence of OPs with high risk of psychological distress and low self-reported well-being.

This tendency was also demonstrated in relation to the operating Regions, with OPs working in the most affected Regions experiencing worst psychosocial conditions. As previously demonstrated by our study group, gender differences are extremely important in the perception of stress, in fact women are more affected by poor well-being and have worse scores than males in the GHQ-12 questionnaire, indicating an increased risk of psychological distress [25]. Previous studies have demonstrated how this phenomenon is influenced by different coping strategies: males tend to adopt problem-focused coping behaviours when confronted with emerging problems while females prefer emotional-focused coping behaviours, which make them more susceptible to mental distress [26].

Lockdown measures, put in place to slow down the spread of the virus, damaged both economies and societies. Our study shows how they also impacted on the mental health of OPs, forced to reorganize their activities in a context of social isolation. The questions of the survey exploring their emotions (“*Do you feel sheltered at home*?*”*, *“Do you feel alone*?*”*, *“Do you feel comfortable at home*?*”*) have been related to their perceived well-being and psychological distress [27, 28].

The results of this study should be considered in the light of some limitations. Firstly, the design of a cross-sectional study is limited in assessing temporal relationship between exposure and outcome, and it cannot demonstrate the existence of a causal relationship between lifestyles and socio-demographic characteristics and measured outcomes. Secondly, since the study is based on a convenience sampling of OPs and on the spontaneous participation of the respondents, the raw results could lack in generalization. Indeed, our results are not immune from possible responding bias, i.e. the risk that the willingness of the OPs to participate in the survey could be directly or indirectly related to their attitude to COVID-19 pandemic. Therefore, it should be considered that a web-based survey is the most rapid and universal method to collect substantial data in a short period (like a lockdown) and the analysis of the sample size is consistent with the generalizability of the data with a certain approximation.

This study emphasizes the need to preserve the well-being and mental health of OPs. The COVID-19 pandemic intervened in the already complicated working conditions of the medical category, in a context of increasing violence against health workers, medical-legal problems, reduction of funds for healthcare systems [29]. Even if there is still no consensus on which preventive measures are the most appropriate to prevent the occurrence of mental disorders among OPs [30], it is urgent to put in place policies of psychological support and well-being preservation for the medical class, not only in terms of the protection of the health of the worker, but also in the interest of the proper functioning of the health system and of the quality of care for citizens.

## Supporting information

S1 Dataset (XLSX)
